# Loss of tricellular tight junction protein LSR promotes cell invasion and migration via upregulation of TEAD1/AREG in human endometrial cancer

**DOI:** 10.1038/srep37049

**Published:** 2017-01-10

**Authors:** Hiroshi Shimada, Shyuetsu Abe, Takayuki Kohno, Seiro Satohisa, Takumi Konno, Syunta Takahashi, Tsubasa Hatakeyama, Chihiro Arimoto, Takuya Kakuki, Yakuto Kaneko, Ken-ichi Takano, Tsuyoshi Saito, Takashi Kojima

**Affiliations:** 1 Departments of Obstetrics and Gynecology, University School of Medicine, Sapporo, Japan; 2 Department of Cell Science, Research Institute for Frontier Medicine, Sapporo Medical University School of Medicine, Sapporo, Japan; 3 Departments of Otolaryngology, Sapporo Medical, University School of Medicine, Sapporo, Japan

## Abstract

Lipolysis-stimulated lipoprotein receptor (LSR) is a unique molecule of tricellular contacts of normal and cancer cells. We investigated how the loss of LSR induced cell migration, invasion and proliferation in endometrial cancer cell line Sawano. mRNAs of amphiregulin (AREG) and TEA domain family member 1 (TEAD1) were markedly upregulated by siRNA-LSR. In endometrial cancer tissues, downregulation of LSR and upregulation of AREG were observed together with malignancy, and Yes-associated protein (YAP) was present in the nuclei. siRNA-AREG prevented the cell migration and invasion induced by siRNA-LSR, whereas treatment with AREG induced cell migration and invasion. LSR was colocalized with TRIC, angiomotin (AMOT), Merlin and phosphorylated YAP (pYAP). siRNA-LSR increased expression of pYAP and decreased that of AMOT and Merlin. siRNA-YAP prevented expression of the mRNAs of AREG and TEAD1, and the cell migration and invasion induced by siRNA-LSR. Treatment with dobutamine and 2-deoxy-D-glucose and glucose starvation induced the pYAP expression and prevented the cell migration and invasion induced by siRNA-LSR. siRNA-AMOT decreased the Merlin expression and prevented the cell migration and invasion induced by siRNA-LSR. The loss of LSR promoted cell invasion and migration via upregulation of TEAD1/AREG dependent on YAP/pYAP and AMOT/Merlin in human endometrial cancer cells.

Tricellular tight junctions (tTJs) form at the convergence of bicellular tight junctions (bTJs) where three epithelial cells meet in polarized epithelia^1^,^2^. Lipolysis-stimulated lipoprotein receptor (LSR) is a novel molecular constituent of tricellular contacts localized in most epithelial tissues and has a barrier function^3^. LSR recruits tricellulin (TRIC), which is the first molecular component of tTJs^1^, and the interaction between the cytoplasmic domain of LSR and the C-terminal cytoplasmic domain of TRIC is required for this recruitment^3^. The LSR-related proteins immunoglobulin-like domain containing receptor ILDR1 and ILDR2 also expressed at tricellular contacts of many epithelial cells and recruit tricellulin, and ILDR1 is responsible for the barrier function^4^.

Several studies have reported that loss of bTJ proteins, including claudins and occludin, enhances tumor progression^5^,^6^,^7^,^8^. Loss of the other TJ protein, coxsackie and adenovirus receptor (CAR), promotes the migration and proliferation of pancreatic cancer cells^9^. Expression of the tTJ protein TRIC is decreased in hepatic fibrolamellar carcinoma and tonsillar squamous cell carcinoma compared to normal tissues^10^,^11^. Well-differentiated pancreatic ductal adenocarcinomas significantly overexpress TRIC as compared with poorly differentiated adenocarcinomas, and TRIC expression in the pancreatic cancer shows a significant negative correlation with the degree of differentiation^12^. Furthermore, TRIC expression in gastric carcinoma cells is negatively regulated by snail-induced epithelial-mesenchymal transition (EMT)^13^. It is thought that the tTJ protein LSR is also associated with tumor progression^14^. Knockdown of LSR increases cell motility and invasion by bladder cancer cells^15^.

More recently, we found that the expression of LSR in human endometrial cancer was decreased together with the malignancy and that the loss of LSR induced cell invasion, migration and proliferation in human endometrial cancer cell line Sawano^16^. We have also reported that downregulation of LSR promotes cell invasion via claudin-1-mediated MMPs in endometrial cancer cells^17^. However, the detailed intracellular signaling mechanisms by which the loss of LSR promotes cell invasion, migration and proliferation in endometrial cancer cells remain unknown.

Removal of the tumor suppressor angiomotin (AMOT)/Merlin from the TJ position induces TEAD/AREG via the Hippo/YAP pathway and then enhances the migration, invasion and proliferation of cancer cells^18^. The Hippo/YAP pathway is a key regulator of organ size and tissue homeostasis and is dysregulated in many human cancers^19^. The development and progression of endometrial cancer are in part attributed to the Hippo/YAP pathway^20^. On the other hand, glucose starvation induces activation of pYAP via AMP-dependent protein kinase (AMPK) and the activation of pYAP prevents transcription of TEAD^21^. Dobutamine is an agonist of the β-adrenergic receptor and G-protein coupled receptor (GPCR), and can induce expression of pYAP^22^,^23^. Furthermore, dobutamine has inhibitory effects on gastric adenocarcinoma cells^24^. Crosstalk between glucose metabolism and the Hippo/YAP pathway is important in tissue maintenance and cancer prevention^21^.

In the present study, we investigated the mechanisms by which the loss of LSR induced cell migration, invasion and proliferation in endometrial cancer. The loss of LSR promoted cell invasion and migration via upregulation of TEAD1/AREG dependent on YAP/pYAP and AMOT/Merlin in human endometrial cancer cells. These complex mechanisms are important for studying the behavior and roles of tTJ proteins in cancers.

## Results

### Expression and localization of LSR, AREG and YAP in endometriosis and endometrial carcinoma

**Figure 1:**
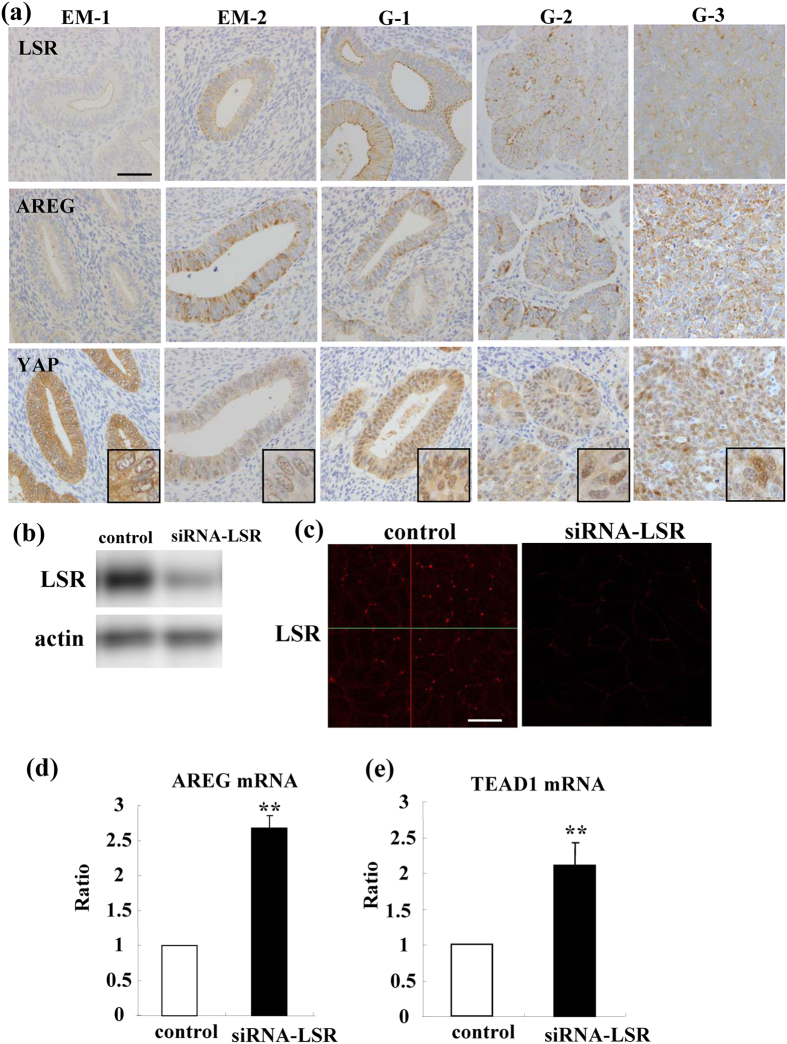
Expression of LSR, AREG and YAP in human endometrial tissues and loss of LSR induces mRNAs of AREG and TEAD1 in Sawano cells. (**a**) Immunohistochemical staining for LSR, AREG and YAP in the tissues of endometriosis (EM) and endometrial cancer (G1-G3). Scale bar: 100 μm. (**b**) Western blotting for LSR in LSR-knockdown Sawano cells. (**c**) Immunocytochemical staining for LSR in LSR-knockdown Sawano cells. Scale bars: 20 μm. (**d**) Real-time PCR for mRNAs of AREG and TEAD1 in LSR-knockdown Sawano cells. The results are shown as bar graphs. Control vs. siRNA-LSR: **p < 0.01.

To investigate the distribution and expression of LSR, AREG and YAP during the carcinogenesis of human endometrial cancer, immunohistchemical staining for LSR, AREG and YAP was performed using paraffin embedded sections of endometriosis and endometrial cancer tissues. In endometriosis LSR was observed not only in the subapical region but also throughout the lateral region and AREG was strongly observed, whereas YAP was observed in cytoplasm but not in nuclei (Fig. 1a). In endometrial cancer which was diagnosed as the classic endometrial type I (endometrioid), LSR and AREG were highly expressed in some cancer cells that formed gland-like structures (Fig. 1a). Furthermore, LSR expression decreased and that of AREG increased in G2 and G3 endometrial cancers compared to G1 (Fig. 1a). YAP was observed in the nuclei of all cancer cells (Fig. 1a).

### Loss of LSR induces expression of mRNAs of AREG and TEAD1 in Sawano cells

To investigate how the loss of LSR induced cell migration, invasion and proliferation in the endometrial cancer cell line Sawano, we first performed DNA microarray analysis of Sawano cells transfected with the siRNA of LSR. In Western blotting and immunocytochemical staining, LSR was found to be decreased by knockdown of LSR (Fig. 1b,c). In the DNA microarray, expression of the mRNAs of AREG and TEAD1 in siRNA-LSR-Sawano cells was markedly increased compared to the control (Table 1). In real-time PCR analysis, expression of the mRNAs of AREG and TEAD1 in siRNA-LSR-Sawano cells was significantly increased compared to the control (Fig. 1d,e).

### siRNA-AREG prevents cell migration and invasion induced by loss of LSR in Sawano cells

**Figure 2:**
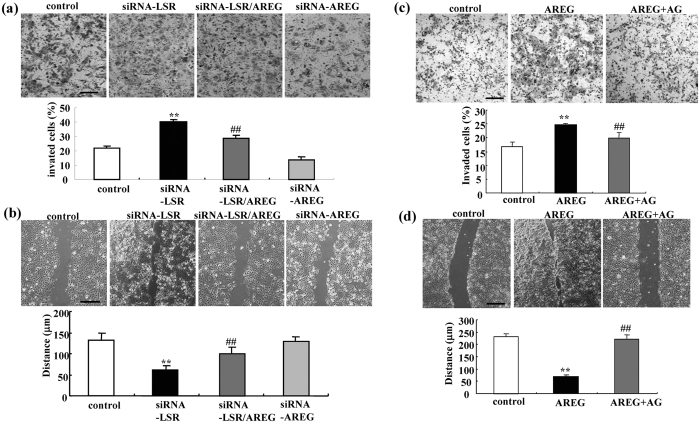
Knockdown of AREG prevents cell invasion and migration induced by loss of LSR and AREG induces cell invasion and migration in Sawano cells. (**a**) Matrigel invasion assay of LSR-knockdown Sawano cells with or without siRNA-AREG. Scale bars: 100 μm. The results are shown as a bar graph. Control vs. siRNA-LSR: **p < 0.01. siRNA-LSR vs. siRNA-LSR + AREG: ^##^p < 0.01. (**b**) Migration assay of LSR-knockdown Sawano cells with and without siRNA-AREG. Scale bars: 400 μm. The results are shown as a bar graph. Control vs. siRNA-LSR: **p < 0.01. siRNA-LSR vs. siRNA-LSR + AREG: ^##^p < 0.01. (**c**) Matrigel invasion assay of AREG-treated Sawano cells with or without AG1478. Scale bars: 100 μm. The results are shown as a bar graph. Control vs. AREG: **p < 0.01. AREG vs. AREG + AG1478: ^##^p < 0.01. (**d**) Migration assay of AREG-treated Sawano cells with and without AG1478. Scale bars: 400 μm. The results are shown as a bar graph. Control vs. AREG: **p < 0.01. AREG vs. AREG + AG1478: ^##^p < 0.01.

To investigate whether autocrine AREG affected the migration and invasion induced by the loss of LSR in Sawano cells, we performed double knockdown of LSR and AREG using the siRNAs. siRNA-AREG prevented the migration and invasion induced by the loss of LSR in Sawano (Fig. 2a,b). It did not affect the cell migration and invasion of control Sawano cells (Fig. 2a,b).

### Treatment with AREG induces cell migration and invasion in Sawano cells

To investigate whether AREG alone affected the migration, invasion and proliferation of control Sawano, the cells were treated with 100 ng/ml recombinant human AREG with or without 10 μM EGFR inhibitor AG1478. Treatment with AREG induced cell migration and invasion, whereas AG1478 prevented the cell migration and invasion induced by AREG (Fig. 2c,d).

### siRNA-YAP or dobutamine prevents expression of mRNAs of AREG and TEAD1, migration, invasion and proliferation induced by loss of LSR in Sawano cells

**Figure 3:**
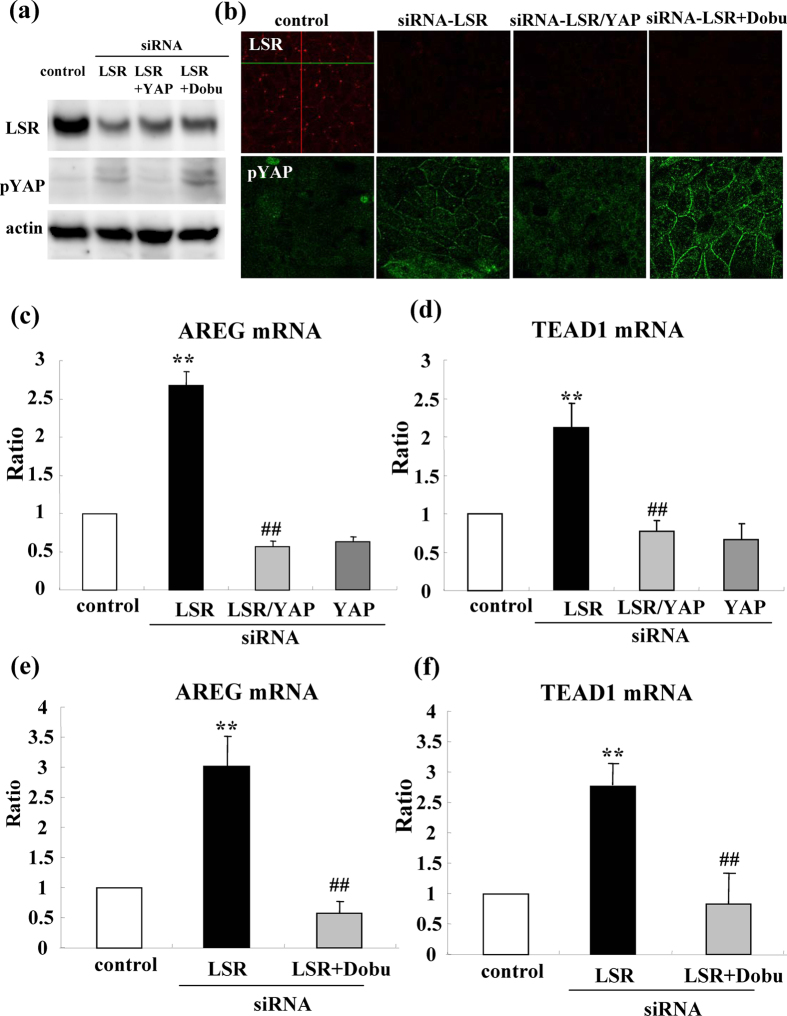
Knockdown of YAP or treatment with dobutamine prevents expression of mRNAs of AREG and TEAD1 induced by loss of LSR. **(a)** Western blotting for LSR and pYAP in LSR-knockdown Sawano cells with or without siRNA-YAP and 10 μM dobutamine. **(b)** Immunocytochemical staining for LSR and pYAP in LSR-knockdown Sawano cells with and without siRNA-YAP or 10 μM dobutamine. Scale bars: 20 μm. **(c–f)** Real-time PCR for mRNAs of AREG and TEAD1 in LSR-knockdown Sawano cells with and without siRNA-YAP or 10 μM dobutamine. The results are shown as bar graphs. Control vs. siRNA-LSR: **p < 0.01. siRNA-LSR vs. siRNA-LSR + YAP and siRNA-LSR + dobutamine: ^##^p < 0.01.

**Figure 4:**
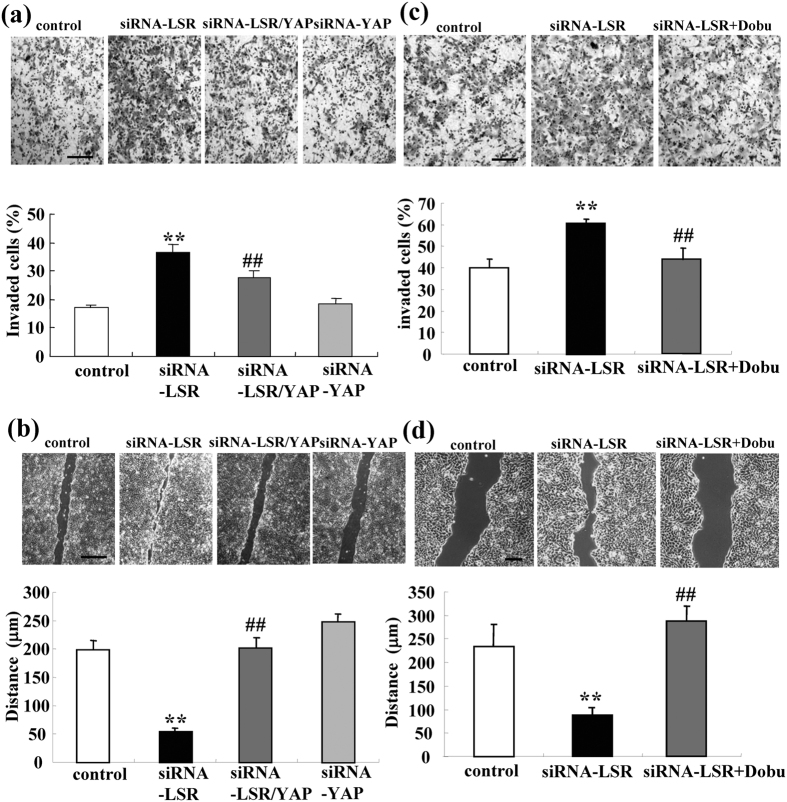
Knockdown of YAP or treatment with dobutamine prevent cell invasion and migration induced by loss of LSR. **(a,c)** Matrigel invasion assay of LSR-knockdown Sawano cells with and without siRNA-YAP or 10 μM dobutamine. Scale bars: 100 μm. The results are shown as bar graphs. Control vs. siRNA-LSR: **p < 0.01. siRNA-LSR vs. siRNA-LSR + YAP or siRNA-LSR + dobutamine: ^##^p < 0.01. **(b,d)** Migration assay of LSR-knockdown Sawano cells with and without siRNA-YAP or 10 μM dobutamine. Scale bars: 400 μm. The results are shown as bar graphs. Control vs. siRNA-LSR: **p < 0.01. siRNA-LSR vs. siRNA-LSR + YAP or siRNA-LSR + dobutamine: ^##^p < 0.01.

It is known that removal of AMOT/Merlin from the TJ position induces TEAD/AREG via the Hippo/YAP pathway and then enhances the migration, invasion and proliferation of cancer cells^18^. Furthermore, dobutamine is a β-adrenergic receptor agonist and can induce pYAP^22^. To investigate whether the Hippo/YAP pathway contributed to the loss of LSR induced mRNAs of AREG and TEAD1, cell migration, invasion and proliferation, we performed double knockdown of LSR and YAP using the siRNAs and pretreatment with 10 μM dobutamine before knockdown of LSR by the siRNA. Western blotting demonstrated that expression of pYAP was induced by siRNA-LSR and that treatment with dobutamine enhanced this expression, whereas siRNA-YAP inhibited the pYAP expression induced by siRNA-LSR (Fig. 3a). In immunocytochemical staining, the pYAP expression induced by siRNA-LSR and treatment with dobutamine, was localized in submembranes (Fig. 3b). siRNA-YAP and dobutamine prevented expression of the mRNAs of AREG and TEAD1 induced by siRNA-LSR (Fig. 3c–f) siRNA-YAP and dobutamine prevented cell migration and invasion induced by siRNA-LSR (Fig. 4a–d). The only siRNA-YAP did not affect expression of the mRNAs of AREG and TEAD1, cell migration or invasion in the control.

### Glucose starvation prevents cell migration and invasion induced by loss of LSR via YAP in Sawano cells

**Figure 5:**
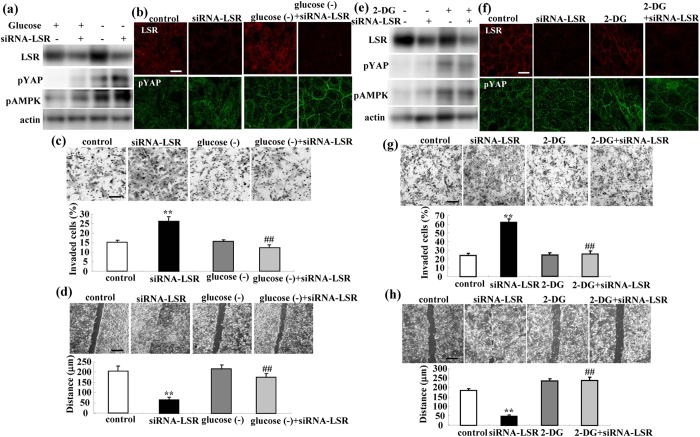
Glucose starvation and treatment with 2-DG induce expression of pYAP and pAMPK and prevent cell invasion and migration induced by loss of LSR. (**a**) Western blotting for LSR, pYAP and pAMPK in LSR-knockdown Sawano cells with and without glucose starvation. (**b**) Immunocytochemical staining for LSR, pYAP and pAMPK in LSR-knockdown Sawano cells with and without glucose starvation. Scale bars: 20 μm. (**c**) Matrigel invasion assay of LSR-knockdown Sawano cells with and without glucose starvation. Scale bars: 100 μm. The results are shown as a bar graph. Control vs. siRNA-LSR: **p < 0.01. siRNA-LSR vs. siRNA-LSR + glucose starvation: ^##^p < 0.01. (**d**) Migration assay of LSR-knockdown Sawano cells with and without glucose starvation. Scale bars: 400 μm. The results are shown as a bar graph. Control vs. siRNA-LSR: **p < 0.01. siRNA-LSR vs. siRNA-LSR + glucose starvation: ^##^p < 0.01. (**e**) Western blotting for LSR, pYAP and pAMPK in LSR-knockdown Sawano cells with and without 2-DG. (**f**) Immunocytochemical staining for LSR, pYAP and pAMPK in LSR-knockdown Sawano cells with and without 2-DG. Scale bars: 20 μm. (**g**) Matrigel invasion assay of LSR-knockdown Sawano cells with or without glucose starvation. Scale bars: 100 μm. The results are shown as a bar graph. Control vs. siRNA-LSR: **p < 0.01. siRNA-LSR vs. siRNA-LSR + 2-DG: ^##^p < 0.01. (**h**) Migration assay of LSR-knockdown Sawano cells with and without 2-DG. Scale bars: 400 μm. The results are shown as a bar graph. Control vs. siRNA-LSR: **p < 0.01. siRNA-LSR vs. siRNA-LSR + 2-DG: ^##^p < 0.01.

Glucose starvation induces activation of pYAP via AMPK and the activation of pYAP prevents the transcription of TEAD^21^. To investigate whether glucose starvation could prevent the expression of the mRNAs of AREG and TEAD1, cell migration, invasion and proliferation induced by the loss of LSR, we performed knockdown of LSR using the siRNA in the medium with and without glucose. Western blotting and immunocytochemical staining demonstrated that glucose starvation induced the expression of pYAP and pAMPK and enhanced that of pYAP and pAMPK induced by siRNA-LSR (Fig. 5a,b). Glucose starvation prevented the cell migration and invasion induced by siRNA-LSR (Fig. 5c,d).

### Treatment with 2-deoxy-D-glucose prevents Sawano cell migration and invasion induced by loss of LSR via YAP

To investigate whether the glucose analog 2-deoxy-D-glucose (2-DG) could prevent the cell migration and invasion induced by loss of LSR, Sawano cells were pretreated with 500 μM 2-DG before knockdown of LSR using the siRNA. Western blotting and immunocytochemical staining showed that treatment with 2-DG induced expression of pYAP and pAMPK (Fig. 5e,f). Pretreatment with 2-DG prevented the cell migration and invasion induced by siRNA-LSR (Fig. 5g,h).

### Distribution and expression of LSR and AMOT in endometriosis and endometrial carcinoma

**Figure 6:**
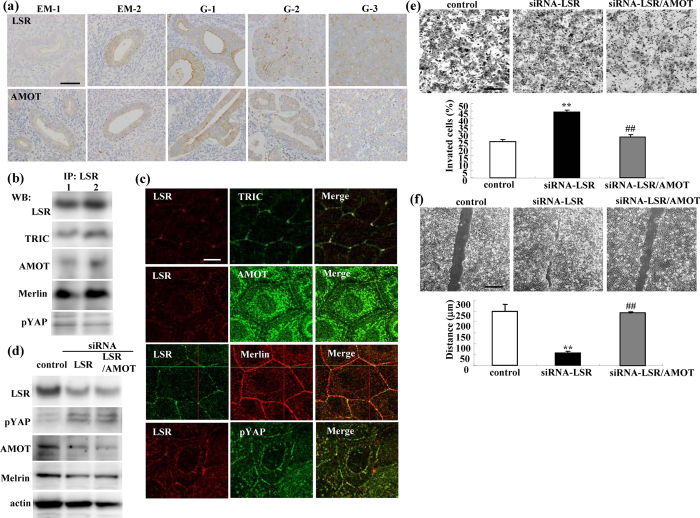
Expression and localization of LSR, AMOT and Merlin in endometrial cancer tissues. Knockdown of AMOT prevents cell invasion and migration induced by loss of LSR. (**a**) Immunohistochemical staining for LSR and AMOT in the tissues of endometriosis (EM) and endometrial cancer (G1-G3). Scale bar: 100 μm. (**b**) Western blotting after coimmunopreciptitation using antibodies of LSR, TRIC, AMOT, Merlin and pYAP in Sawano cells. (**c**) Double immunocytochemical staining for LSR and TRIC, LSR and AMOT, LSR and Merlin, LSR and pYAP in Sawano cells. Scale bars: 10 μm. (**d**) Western blotting for LSR, pYAP, AMOT and Merlin in LSR-knockdown Sawano cells with and without siRNA-AMOT. (**e**) Matrigel invasion assay of LSR-knockdown Sawano cells with and without siRNA-AMOT. Scale bars: 100 μm. The results are shown as a bar graph. Control vs. siRNA-LSR: **p < 0.01. siRNA-LSR vs. siRNA-LSR + AMOT: ^##^p < 0.01. (**f**) Migration assay of LSR-knockdown Sawano cells with and without siRNA-AMOT. Scale bars: 400 μm. The results are shown as a bar graph. Control vs. siRNA-LSR: **p < 0.01. siRNA-LSR vs. siRNA-LSR + AMOT: ^##^p < 0.01.

To investigate the relationship between AMOT and LSR during the carcinogenesis of human endometrial cancer, immunohistchemical staining for LSR and AMOT was performed using paraffin embedded sections of endometriosis and endometrial cancer tissues. In endometriosis, LSR and AMOT were observed not only in the subapical region but also throughout the lateral region (Fig. 6a). In endometrial cancer diagnosed as the classic endometrial type I (endometrioid), LSR and AMOT were highly expressed in some cancer cells that formed gland-like structures (Fig. 6a). Furthermore, the expression of LSR and AMOT was decreased in G2 and G3 endometrial cancers compared to G1 (Fig. 6a).

### Distribution and expression of LSR, AMOT, Merlin and pYAP in Sawano cells with and without siRNA-LSR

To investigate whether the loss of LSR affected endometrial cancer malignancy via AMOT/Merlin, we examined the relationships among LSR, AMOT/Merlin and pYAP in Sawano cells. In Western blotting after coimmunopreciptitation using antibodies of LSR, TRIC, AMOT, Merlin and pYAP were detected in Sawano cells (Fig. 6b). In immunocytochemical staining, AMOT, Merlin and pYAP were localized at bTJ areas and in part colocalized with LSR in tTJ areas, whereas only TRIC was colocalized with LSR in those areas (Fig. 6c). siRNA-LSR increased pYAP expression and decreased expression of AMOT and Merlin (Fig. 6d). siRNA-AMOT enhanced the decrease of AMOT expression induced by siRNA-LSR (Fig. 6d).

### siRNA-AMOT prevents cell migration and invasion induced by loss of LSR in Sawano cells

To investigate whether AMOT affected the Sawano cell migration, invasion and proliferation induced by loss of LSR, we performed double knockdown of LSR and AMOT. siRNA-AMOT prevented the cell migration and invasion induced by the loss of LSR in Sawano cells (Fig. 6e,f).

### siRNA-AMOT by itself induces cell migration and invasion via YAP in Sawano cells

**Figure 7:**
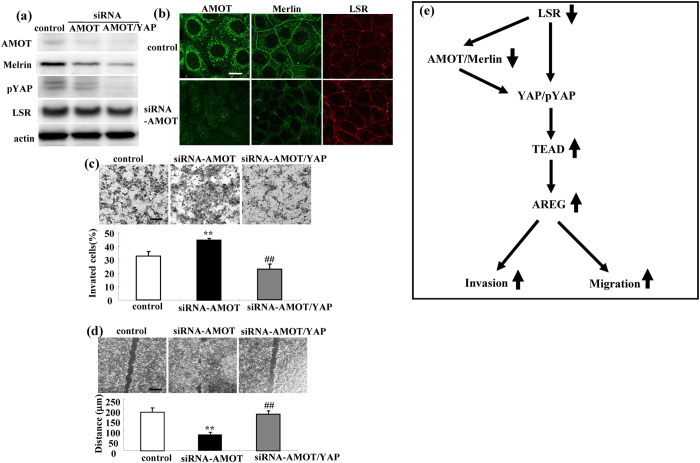
Knockdown of AMOT induces Sawano cell invasion and migration via YAP. **(a)** Western blotting for AMOT, Merlin, pYAP and LSR in AMOT-knockdown Sawano cells with and without siRNA-YAP. **(b)** Immunocytochemical staining for AMOT, Merlin and LSR in AMOT-knockdown Sawano cells. Scale bars: 10 μm. **(c)** Matrigel invasion assay of AMOT-knockdown Sawano cells with and without siRNA-YAP. Scale bars: 100 μm. The results are shown as a bar graph. Control vs. siRNA-AMOT: **p < 0.01. siRNA-AMOT vs. siRNA-AMOT + YAP: ^##^p < 0.01. **(d)** Migration assay of AMOT-knockdown Sawano cells with and without siRNA-YAP. Scale bars: 400 μm. The results are shown as a bar graph. Control vs. siRNA-AMOT: **p < 0.01. siRNA-AMOT vs. siRNA-AMOT + YAP: ^##^p < 0.01. **(e)** Overview of the intracellular signal mechanisms by which the loss of LSR promotes cell invasion and migration via upregulation of TEAD/AREG in human endometrial cancer cells.

To investigate whether AMOT by itself affected the cell migration, invasion and proliferation of Sawano cells via YAP, we performed double knockdown of AMOT and YAP using the siRNAs. siRNA-AMOT decreased the expression of Merlin and pYAP but not that of LSR, and siRNA-YAP enhanced the former effects (Fig. 7a,b). siRNA-AMOT induced the migration and invasion of Sawano cells and siRNA-YAP prevented the cell migration and invasion induced by siRNA-AMOT (Fig. 7c,d).

## Discussion

In the present study, we first found that the loss of tricellular tight junction protein LSR promoted the invasion and migration of human endometrial cancer cells via upregulation of TEAD/AREG.

The Hippo/YAP pathway is a key regulator of organ size and tissue homeostasis and is dysregulated in many human cancers^19^. YAP acts to stimulate expression of genes that promote cell proliferation and inhibit apoptosis via TEAD family transcription factors^25^. The epidermal growth factor receptor (EGFR) ligand AREG, as a transcriptional target of YAP, contributes to YAP-mediated cell proliferation and migration^26^. Furthermore, removal of the tumor suppressor AMOT/Merlin from the TJ position induces TEAD/AREG via the Hippo/YAP pathway and then enhances the migration, invasion and proliferation of cancer cells^18^. The development and progression of endometrial cancer are also attributed to the Hippo/YAP pathway^20^.

In the present study, in human endometrial cancer tissues, downregulation of LSR and upregulation of AREG were observed together with malignancy. In human endometrial cancer cell line Sawano, mRNAs of AREG and TEAD1 were markedly upregulated by the loss of LSR. Furthermore, siRNA-AREG prevented the cell migration and invasion induced by the loss of LSR. AREG treatment induced cell migration and invasion and the inhibitor of EGF receptor AG1478 prevented them. These findings suggested that the loss of LSR from the tTJ position induced TEAD/AREG and then enhanced the migration and invasion of cancer cells like AMOT/Merlin.

The bTJ proteins ZO-2 and YAP2 colocalize in the nucleus and the ZO-2-YAP2 complex increases cell proliferation^27^. The TJ protein PARD3 forms a polarity complex with PAR6 and aPKC, and activates YAP/TAZ to promote cell growth^28^. The bTJ protein occludin is also colocalized with Hippo/YAP pathway elements^29^. The loss of occludin enhances tumor progression via cell migration and invasion^8^. In the present study, in human endometrial cancer tissues, YAP was positive in the nuclei of all cancer cells. In Sawano cells, LSR was colocalized with pYAP at tricellular contacts and it was detected in coimmunopreciptitates using the pYAP antibody. The loss of LSR increased the expression of pYAP, and siRNA-YAP prevented expression of not only mRNAs of AREG and TEAD1 but also the cell migration and invasion induced by the loss of LSR. Treatment with the GPCR agonist dobutamine induced the expression of pYAP and prevented the expression of AREG mRNA, cell migration and invasion induced by loss of LSR. Furthermore, treatment with the non-metabolizable glucose analog 2-deoxy-D-glucose and glucose starvation induced expression of pYAP and pAMPK and prevented the cell migration and invasion induced by the loss of LSR. Crosstalk between glucose metabolism and the Hippo/YAP pathway is important in tissue maintenance and cancer prevention^21^. AMPK-induced YAP inhibition can suppress oncogenic transformation via the Hippo/YAP pathway^30^. GPCR signaling can either activate or inhibit the Hippo/YAP pathway^31^. These findings suggested that the loss of LSR promoted cell invasion and migration via upregulation of TEAD/AREG via the Hippo/YAP pathway in human endometrial cancer cells, although the mechanism by which this loss of LSR increased expression of pYAP was unclear in the present study.

Dobutamine has inhibitory effects on gastric adenocarcinoma cells^24^. In the present study, to investigate whether dobutamine by itself affected the cell migration and invasion of endometrial cancer Sawano cells, the cells were treated with dobutamine. This treatment induced expression of pYAP and inhibited cell migration and invasion (Supplemental Fig. 1). Suggesting that dobutamine might be useful not only for treatment of gasntric cancer, but also for endometrial cancer.

On the other hand, in Sawano cells, LSR was also colocalized with TRIC, AMOT and Merlin at tricellular contacts and it was detected in immunopreciptitates using their antibodies. siRNA-LSR decreased the expression of AMOT and Merlin. siRNA-AMOT decreased that of Merlin and prevented the cell migration and invasion induced by the loss of LSR, while siRNA-AMOT by itself induced cell migration and invasion via YAP. The loss of AMOT/Merlin enhances the cell migration, invasion and proliferation of cancer cells via the Hippo/YAP pathway^18^. It also results in aberrant activation of Wnt/β-catenin signaling^32^. AMOT is highly expressed in breast cancer and promotes the cancer cell proliferation and invasion^33^. Thus it appears that the loss of LSR in part promoted cell invasion and migration via AMOT/Merlin in human endometrial cancer cells. However, it is necessary to investigate the relationship between LSR and AMOT/Merlin at tricellular contacts and the roles of AMOT/Merlin in human endometrial cancer cells in more detail.

Taken together, our findings indicated that the loss of LSR promoted cell invasion and migration via upregulation of AREG dependent on YAP/pYAP and AMOT/Merlin in human endometrial cancer cells (Fig. 7e). Furthermore, we also found that loss of LSR promoted cell invasion of human endometrial cancer via claudin-1-mediated matrix metalloproteinases^17^. These complex mechanisms are important for studying the behavior and the roles of tTJ proteins in cancers. Leptin promotes human endometriotic cell migration and invasion^34^. We previously reported that, in Sawano cells, a decrease of LSR expression induced by leptin and an increase of it induced by adiponectin and AMPK activators of the drugs for type 2 diabetes metformin and berberine, were observed and that the AMPK activators prevented cell migration and invasion induced by loss of LSR by leptin treatment^16^. It is possible that metformin and berberine may prevent the cell migration and invasion induced by the loss of LSR via AMPK/YAP. AMPK, a sensor of cellular energy stress may contribute to promote the cancer malignancy induced by the loss of LSR.

## Materials and Methods

### Ethics statement

The protocol for human study was reviewed and approved by the ethics committee of the Sapporo Medical University School of Medicine. Written informed consent was obtained from each patient who participated in the investigation. All experiments were carried out in accordance with the approved guidelines and with the Declaration of Helsinki.

### Reagents and Antibodies

A rabbit polyclonal anti-actin antibody, EGFR inhibitor (AG1478) and 2-deoxy-D-glucose (2-DG) were obtained from Sigma-Aldrich (St. Louis, Mo., USA). Dobutamine hydrochloride was obtained from Wako Laboratory Chemicals (Tokyo, Japan). Recombinant human amphiregulin was obtained from PeproTech (Rocky Hill, NJ). Rabbit polyclonal anti-lipolysis-stimulated receptor (LSR) and anti-tricellulin (TRIC) antibodies were obtained from Zymed Laboratories (San Francisco, CA). A mouse monoclonal anti-LSR antibody was obtained from Abnova (Yaipei, Taiwan). A rabbit polyclonal anti-angiomotin (AMOT) antibody was obtained from Bioss, Inc. (Woburn, MA). Rabbit monoclonal anti-Merlin, anti-phospho- YAP (ser127) and anti-phospho-AMPK (ther172) antibodies were obtained from Cell Signaling Technology Japan (Tokyo, Japan). A rabbit polyclonal anti-YAP antibody was obtained from Novus Biologicals (Littleton, CO). Alexa 488 (green)-conjugated anti-rabbit IgG and Alexa 594 (red)-conjugated anti-mouse IgG antibodies were purchased from Molecular Probes, Inc. (Eugene, OR).

### Cell line culture and treatment

The human endometrioid endometrial cancer cell line Sawano (RCB1152) was purchased from RIKEN Bio-Resource Center (Tsukuba, Japan). The cells were maintained with MEM (Sigma-Aldrich) supplemented with 10% dialyzed fetal bovine serum (FBS; Invitrogen, Carlsbad, CA, USA). The medium contained 100 U/ml penicillin, 100 μg/ml streptomycin and 50 μg/ml amphotericin-B. Sawano cells were plated on 35- and 60-mm culture dishes, which were coated with rat tail collagen (500 μg dried tendon/ml in 0.1% acetic acid) and incubated in a humidified 5% CO_2_ incubator at 37 °C. Sawano cells were treated with 100 ng/ml amphiregulin with or without 10 μM AG1478.

### RNA interference and transfection

siRNA duplex oligonucleotides against LSR (forward sense 5′-CCCACGCAACCCAUCGUCAUCUGGA-3′, reverse sense 5′-UCCAGAUGACGAUGGGUUGCGUGGG-3′) were synthesized by Thermo Fisher Scientific (Waltham, MA). siRNA duplex oligonucleotides against AMOT (sc-72489), YAP (sc-38637) and AREG (sc-39412) were synthesized by SantaCruz Biotechnology, Inc. (Santa Cruz, CA). A scrambled siRNA sequence (BLOCK-iT Alexa Fluor fluorescent, Invitrogen) was employed as control siRNA. At 24 h after plating, Sawano cells were transfected with 100 nM siRNAs of LSR, AREG, YAP and AMOT using Lipofectamine^TM^ RNAiMAX Reagent (Invitrogen). Some cells were transfected with 100 nM siRNA of LSR with or without 10 μM dobutamine, 500 μM 2-DG and glucose-free medium (Glucose free MEM, Sigma-Aldrich).

### Immunohistochemical analysis

Human endometrial carcinoma tissues and human endometriomal tissues were obtained from 6 patients with endometriosis and 15 patients with endometrial adenocarcinoma (G1: 7, G2: 4, and G3: 4) who underwent hysterectomy at Sapporo Medical University Hospital. Written informed consent was obtained from all patients. The study was approved by the ethics committee of Sapporo Medical University. Hematoxylin and eosin-stained slides from each case were reviewed, and the diagnosis and grades of the tumors were determined according to the guidelines of the WHO classification. The diagnoses of endometriosis and endometrial adenocarcinomas were established by both gynecologists and pathologists. All endometrial adenocarcinoma were the classic endometrial type I.

Human endometriosis and endometrial cancer tissues were embedded in paraffin after fixation with 10% formalin in PBS. Briefly, 5-μm-thick sections were dewaxed in xylene, rehydrated in ethanol, and heated with Vision BioSystems Bond Max using ER2 solution (Leica) in an autoclave for antigen retrieval. Endogenous peroxidase was blocked by incubation with 3% hydrogen peroxide in methanol for 10 min. The tissue sections were then washed twice with Tris-buffered saline (TBS) and preblocked with Block Ace for 1 h. After washing with TBS, the sections were incubated with anti-LSR (1:100), anti-AREG (1:300), anti-YAP (1:100) and anti-AMOT (1:100) antibodies for 1 h. The sections were then washed three times in TBS and incubated with Vision BioSystems Bond Polymer Refine Detection kit DS9800. After three washes in TBS, a diamino-benzidine tetrahydrochloride working solution was applied. Finally, the sections were counterstained with hematoxylin.

### Immunocytochemical staining

Cells cultured in 35-mm glass-coated wells (Iwaki, Chiba, Japan) were fixed with cold acetone and ethanol (1:1) at −20 °C for 10 min. After rinsing in PBS, the cells were incubated with anti-LSR (1:100), anti-pYAP (1:100), anti-AMOT (1:100), and anti-Merlin (1:100) antibodies at room temperature for 1 h. Alexa Fluor 488 (green)-conjugated anti-rabbit IgG and Alexa Fluor 594 (red)-conjugated anti-mouse IgG (Invitrogen) were used as secondary antibodies. The specimens were examined using an epifluorescence microscope (Olympus, Tokyo, Japan) and a confocal laser scanning microscope (LSM5; Carl Zeiss, Jena, Germany).

### GeneChip analysis

Microarray slides were scanned using a 3D-GENE human Oligochip 25k. (TORAY, Tokyo, Japan) and microarray images were automatically analyzed using AROS^TM^, version 4.0 (Operon Biotechnologies, Tokyo, Japan).

### RNA isolation and real-time PCR analysis

Total RNA was extracted and purified using TRIzol (Invitrogen, Carlsbad, CA). One microgram of total RNA was reverse-transcribed into cDNA using a mixture of oligo (dT) and Superscript II reverse transcriptase according to the manufacturer’s recommendations (Invitrogen). Real-time PCR detection was performed using a TaqMan Gene Expression Assay kit with a StepOnePlus^TM^ real-time PCR system (Applied Biosystems, Foster City, CA). The amount of 18 S ribosomal RNA (rRNA) (Hs99999901) mRNA in each sample was used to standardize the quantity of the mRNAs of AREG (Hs00950669) and TEAD1 (Hs00933391). The relative mRNA-expression levels of the control and treated samples were calculated by the difference of the threshold cycle (comparative C_T_ [∆∆C_T_] method) and presented as the average of triplicate experiments with a 95% confidence interval.

### Western blot analysis

The cultured cells were scraped from 60 mm dishes containing 400 μl of buffer (1 mM NaHCO^3^ and 2 mM phenylmethylsulfonyl fluoride), collected in microcentrifuge tubes, and then sonicated for 10 s. The protein concentrations of the samples were determined using a BCA protein assay regent kit (Pierce Chemical Co.; Rockford, IL, USA). Aliquots of 15 μl of protein/lane for each sample were separated by electrophoresis in 5–20% SDS polyacrylamide gels (Wako, Osaka, Japan), and electrophoretically transfered to a nitrocellulose membrane (Immobilon; Millipore Co.; Bedford, UK). The membrane was saturated with blocking buffer (25 mM Tris, pH 8.0, 125 mM NaCl, 0.1% Tween 20, and 4% skim milk) for over 30 min at room temperature and incubated with anti-LSR (1:1000), anti-tricellulin (1:1000), anti-AMOT (1:500), anti-Merlin (1:500), anti-pYAP (1:500) and anti-actin (1:1000) antibodies at room temperature for over 1 h. Then it was incubated with HRP-conjugated anti-mouse and anti-rabbit IgG antibodies at room temperature for 1 h. The immunoreactive bands were detected using an ECL Western blot system.

### Coimmunopreciptitation

The dishes were washed with PBS twice and 300 μl of NP-40 lysis buffer (50 mM Tris–HCl, 2% NP-40, 0.25 mM Na-deoxycholate, 150 mM NaCl, 2 mM EGTA, 0.1 mM Na3VO4, 10 mM NaF, 2 mM PMSF) was added to the 60-mm dishes. The cells were scraped off, collected in microcentrifuge tubes and then sonicated for 10 s. Cell lysates were incubated with protein A-Sepharose CL-4B (Pharmacia LKB Biotechnology, Uppsala, Sweden) for 1 h at 4 °C and then clarified by centrifugation at 15,000 g for 10 min. The supernatants were incubated with the polyclonal anti-LSR antibody bound to protein A-Sepharose CL-4B overnight at 4 °C. After incubation, immunoprecipitates were washed extensively with the same lysis buffer and subjected to Western blot analysis with anti-LSR, anti-tricellulin, anti-AMOT, anti-Merlin and anti-pYAP antibodies.

### Matrigel invasion assay

For the invasion assay, we used Matrigel (Becton Dickinson Labware, Bedford, MA) and Cell Culture Insert (pore size 8 μm; Becton Dickinson Labware). Sawano cells were plated onto the upper chamber coated with Matrigel and the lower chamber of the Transwell was filled with human fibroblast conditioned medium containing 10 nM EGF as an adhesive substrate. Then the cells were incubated for 36 h, after which the upper chamber was fixed with 100% methanol for 10 min and stained with Giemsa for 20 min. The areas of invading cells were measured using a microscope imaging system (Olympus, Tokyo, Japan).

### Migration assay

After the Sawano cells were plated onto the 35 mm dishes, they were cultured to confluence. At 24 h we wounded the cell layer using a plastic pipette tip (P200), and measured the length of the wound by using a microscope imaging system (Olympus, Tokyo, Japan).

### Data analysis

Each set of results shown is representative of at least three separate experiments. Results are given as means ± SEM. Differences between groups were tested by ANOVA followed by a post-hoc test and an unpaired two-tailed Student’s *t* test.

## Additional Information

**How to cite this article**: Shimada, H. *et al*. Loss of tricellular tight junction protein LSR promotes cell invasion and migration via upregulation of TEAD1/AREG in human endometrial cancer. *Sci. Rep.* **7**, 37049; doi: 10.1038/srep37049 (2017).

**Publisher's note:** Springer Nature remains neutral with regard to jurisdictional claims in published maps and institutional affiliations.

## Supplementary Material

Supplemental Figure 1

## Figures and Tables

**Table 1:** List of gene probes which are up-regulated in Sawano cells transfected with siRNA-LSR.

Gene name	ID	Gene Bank ID	Fold-change control vs siRNA-LSR
AREG	AHsV10001850	NM_001657.2	3.3
AREGB	CHsGV10000152	NM_001657	12.71
TEAD1	H300019821	NM_021961	2.7
