# Supplementary material for: Loss of tricellular tight junction protein LSR promotes cell invasion and migration via upregulation of TEAD1/AREG in human endometrial cancer
Source: Sci Rep. 2017 Jan 10;7:37049. doi: 10.1038/srep37049 (PMC5223122; doi:10.1038/srep37049)
Supplement: Supplemental Figure 1 [file srep37049-s1.pdf]

Loss of tricellular tight junction protein LSR promotes cell invasion and migration via upregulation of TEAD1/AREG in human endometrial cancer

Hiroshi Shimada[1,3], Shyuetsu Abe[1,3#], Takayuki Kohno[3],
Seiro Satohisa[1], Takumi Konno[3], Syunta Takahashi[3],
Tsubasa Hatakeyama[3], Chihiro Arimoto[3], Takuya Kakuki[2,3],
Yakuto Kaneko[2,3], Ken-ichi Takano[2], Tsuyoshi Saito[1]
and Takashi Kojima[3*]

Departments of [1]Obstetrics and Gynecology, and [2]Otolaryngology,
Sapporo Medical University School of Medicine, Sapporo, Japan
Department of [3]Cell Science, Research Institute for Frontier Medicine,
Sapporo Medical University School of Medicine, Sapporo, Japan

#: equal first authors
*Author for correspondence: Takashi Kojima, Ph.D.
Department of Cell Science, Research Institute for Frontier Medicine,
Sapporo Medical University School of Medicine, South-1, West-17,
Chuo-ku, Sapporo 060-8556, Japan
Tel: +81-11-688-9612
Fax: +81-11-611-2299
E-mail address: ktakashi@sapmed.ac.jp

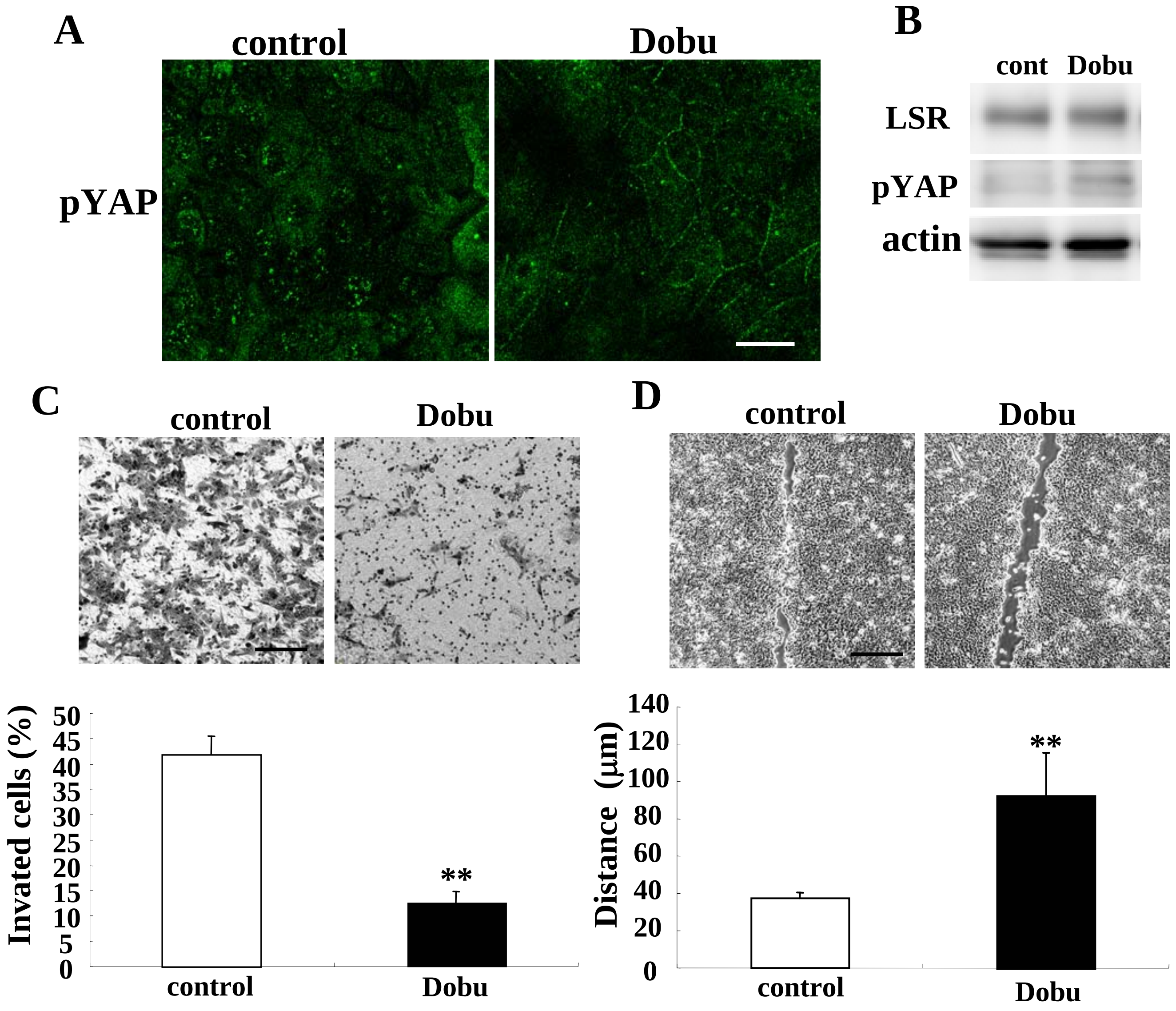

Supplemental Fig.1 Treatment with dobutamine inhibits Sawano cell invasion and migration
A. Immunocytochemical staining for pYAP in Sawano cells treated with 10 μM dobutamine. Scale bars: 20 μm. **B.** Western blotting for LSR and pYAP in Sawano cells treated with 10 μM dobutamine (Dobu). **C.** Matrigel invasion assay of Sawano cells treated with 10 μM dobutamine (Dobu). Scale bars: 100 μm. The results are shown as a bar graph. Control vs. dobutamine: **p<0.01. **D.** Migration assay of Sawano cells treated with 10 μM dobutamine (Dobu). Scale bars: 400 μm. The results are shown as a bar graph. Control vs.dobutamine: **p<0.01.
